# Supplementary figures and images for: A versatile new tool derived from a bacterial deubiquitylase to detect and purify ubiquitylated substrates and their interacting proteins
Source: PLoS Biol. 2022 Jun 30;20(6):e3001501. doi: 10.1371/journal.pbio.3001501 (PMC9278747; doi:10.1371/journal.pbio.3001501)

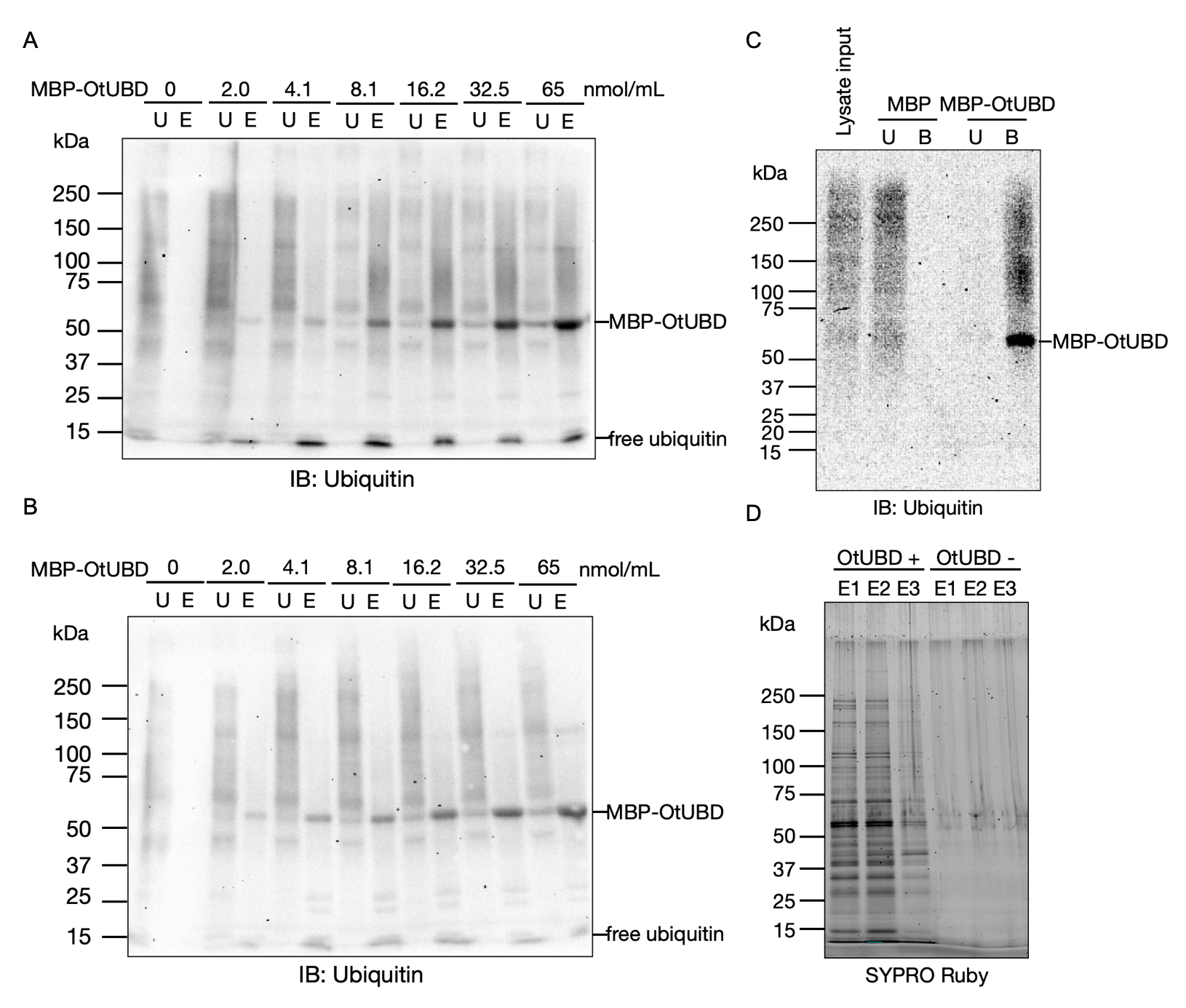

Supplement: S1 Fig — (A, B) Ubiquitin pulldowns with different amounts of MBP-OtUBD. In A, the pulldown was performed by first binding MBP-OtUBD to an amylose resin and then incubating the resin with yeast cell lysate. In B, pulldown was performed by incubating the lysate with MBP-OtUBD and then binding the complexes to amylose resin. U, unbound fraction; E, fraction eluted with maltose. The bands seen at the expected molecular mass of MBP-UBD were likely a result of antibody cross-reactivity. (C) Anti-ubiquitin blot of MBP-OtUBD pulldowns from HEK293T whole cell lysates. U, unbound fraction; B, bound fraction (eluted with SDS sample buffer). (D) SYPRO Ruby protein stain of the eluates from OtUBD-resin in Fig 2B. E1/E2/E3, eluted fractions from serial low pH elutions. MBP, maltose-binding protein. (TIF) [file pbio.3001501.s001.tif]

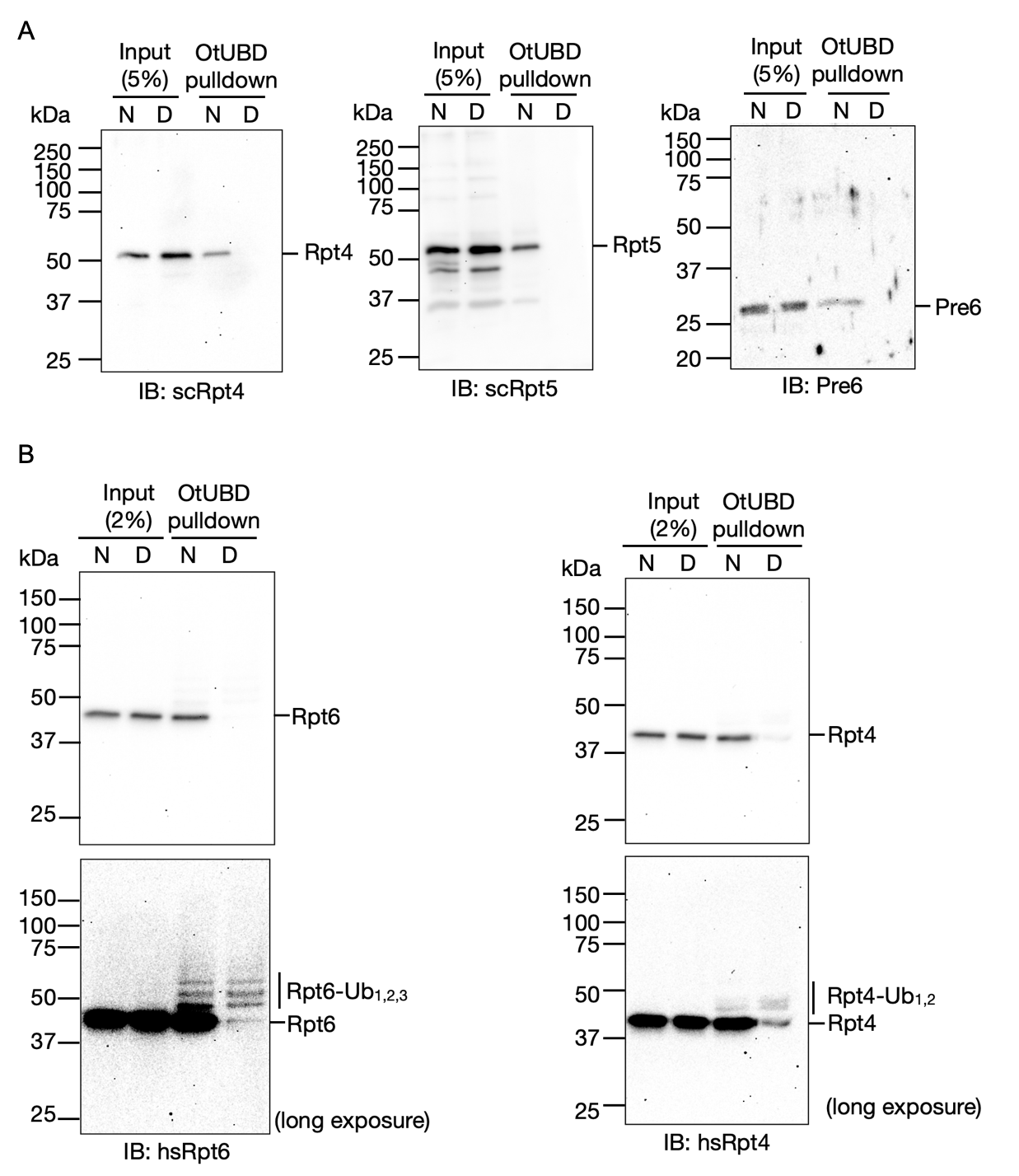

Supplement: S2 Fig — (A) Western blots of yeast proteasomal subunits in OtUBD pulldown samples. Unmodified yeast proteasomal subunits (Rpt4, Rpt5, and Pre6) bound to the OtUBD resin under native conditions but not following denaturation of the lysate prior to pulldown. N, Native condition; D, Denaturing condition. (B) Western blots of human proteasomal subunits in OtUBD pulldown samples. Unmodified human proteasomal subunit Rpt6 and Rpt4 bound strongly to OtUBD resin under native conditions but only weakly following extract denaturation. Modified Rpt6 and Rpt4 (likely ubiquitylated) bound to the OtUBD resin under both native and denaturing conditions. N, Native condition; D, Denaturing condition. (TIF) [file pbio.3001501.s002.tif]

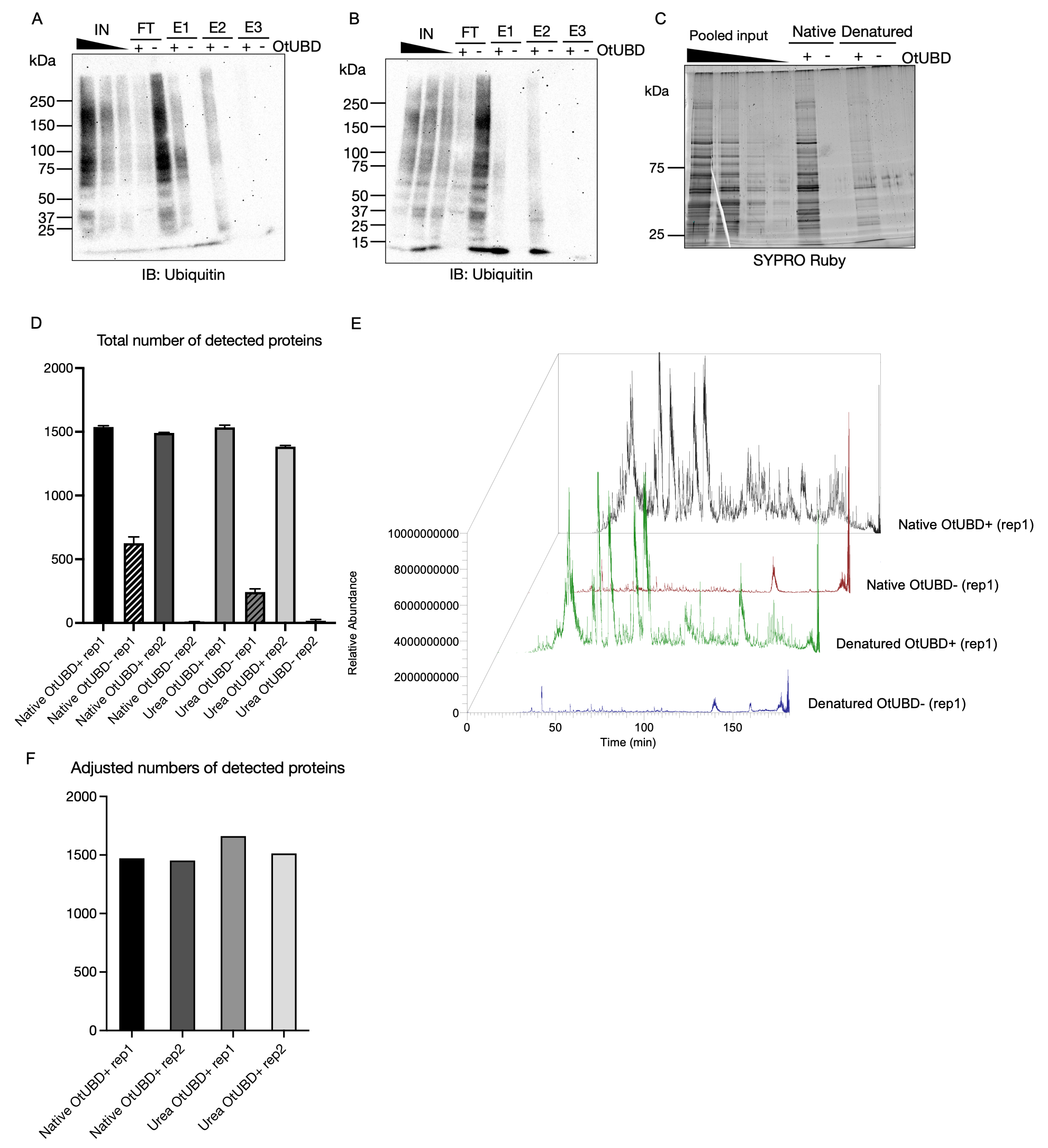

Supplement: S3 Fig — (A) Representative anti-ubiquitin western blot of OtUBD pulldowns (under native conditions) used for proteomics analysis. IN, input; FT, flow-through; E1/E2/E3, eluted fractions from a series of low pH elutions. (B) Representative anti-ubiquitin blot of OtUBD pulldown samples following extract denaturation (urea) and used for proteomics analysis. (C) Representative SYPRO Ruby protein stain of OtUBD eluates resolved by SDS-PAGE. (D) Number of proteins detected in each biological replicate of OtUBD pulldown-MS and negative control. Error bar represents difference among technical replicates. (E) Overlay of TIC chromatographs of representative OtUBD pulldowns and negative control samples. The negative controls overall have much less peptide spectra compared to the OtUBD pulldown samples. This figure is generated with Thermo Xcalibur Qual Browser (v3.0.63) using.raw files from the corresponding runs (QEp21-2054_Zhang_A1_Native_UBD_pos, QEp21-2050_zhang_a2_native_ubd_neg, QEp21-2036_zhang_a3_denatured_ubd_pos, QEp21-2032_zhang_a4_denatured_ubd_neg), which have been deposited to the ProteomeXchange Consortium and made available to the public (see the Methods section for details). (F) Adjusted number of proteins detected in each biological replicate of OtUBD pulldowns. Only proteins whose TIC value are at least 20 times higher in the OtUBD pulldown samples compared to the corresponding negative control samples are included. (TIF) [file pbio.3001501.s003.tif]

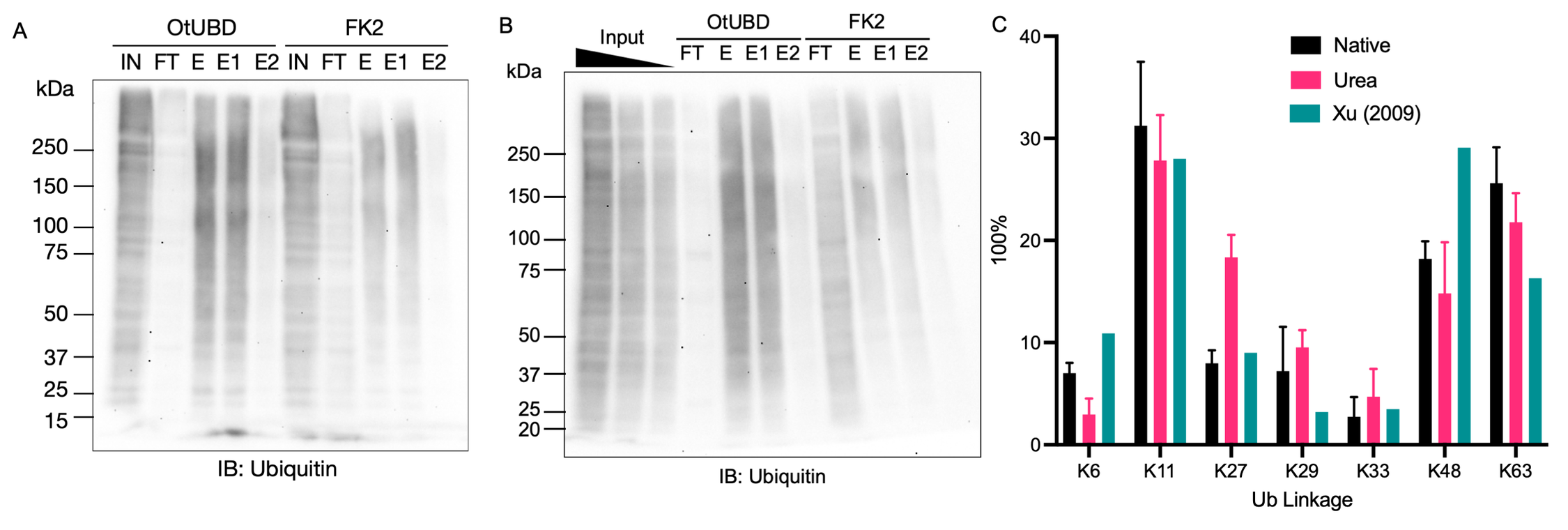

Supplement: S4 Fig — (A, B) Anti-ubiquitin western blot of OtUBD pulldowns, and FK2 antibody IPs used for proteomics analysis. OtUBD and FK2 used in A and B are both from different batches. IN, input; FT, flow-through; E, pooled eluted fractions; E1/E2, eluted fractions from a series of low pH elutions. (C) Quantitation (estimated from total spectral counts) of different ubiquitin linkages under native and denaturing (urea) conditions in the BY4741 yeast ubiquitylome from this study. Comparison was made with quantitative data published in Xu and colleagues. The numeric values supporting this panel can be found in S3 Data. (TIF) [file pbio.3001501.s004.tif]

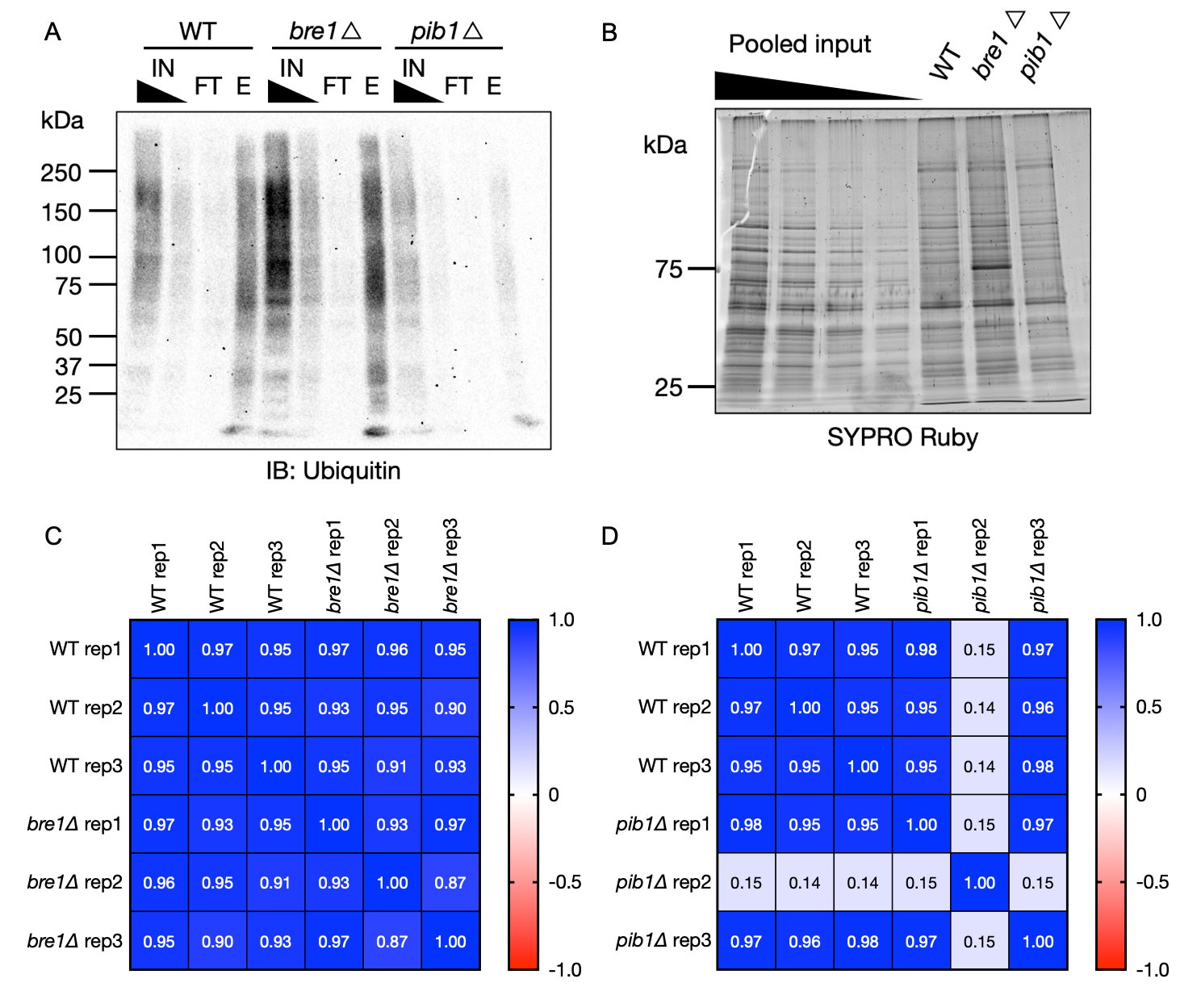

Supplement: S5 Fig — (A) Representative anti-ubiquitin blot of OtUBD pulldowns from WT, bre1△ and pib1△ yeast lysates used for proteomics analysis. IN, input; FT, flow-through; E, pooled eluted fractions. (B) Representative SYPRO Ruby gel showing the total proteins in eluates from OtUBD pulldown from WT, bre1△, and pib1△ yeast lysates. (C, D) Pearson correlation coefficients were calculated between each sample in the analyzed groups using normalized total TIC. Because ubiquitin is present in exceptionally high levels compared to all other proteins, it was excluded from the dataset for this analysis. With the exception of one pib1Δ sample, correlations between different samples were generally high, as expected if the majority of the ubiquitylome was not affected by deletion of a single E3. The low correlation in the single pib1Δ sample was likely due to an error during sample preparation, so the results were excluded from the quantitation. WT, wild-type. (TIF) [file pbio.3001501.s005.tif]

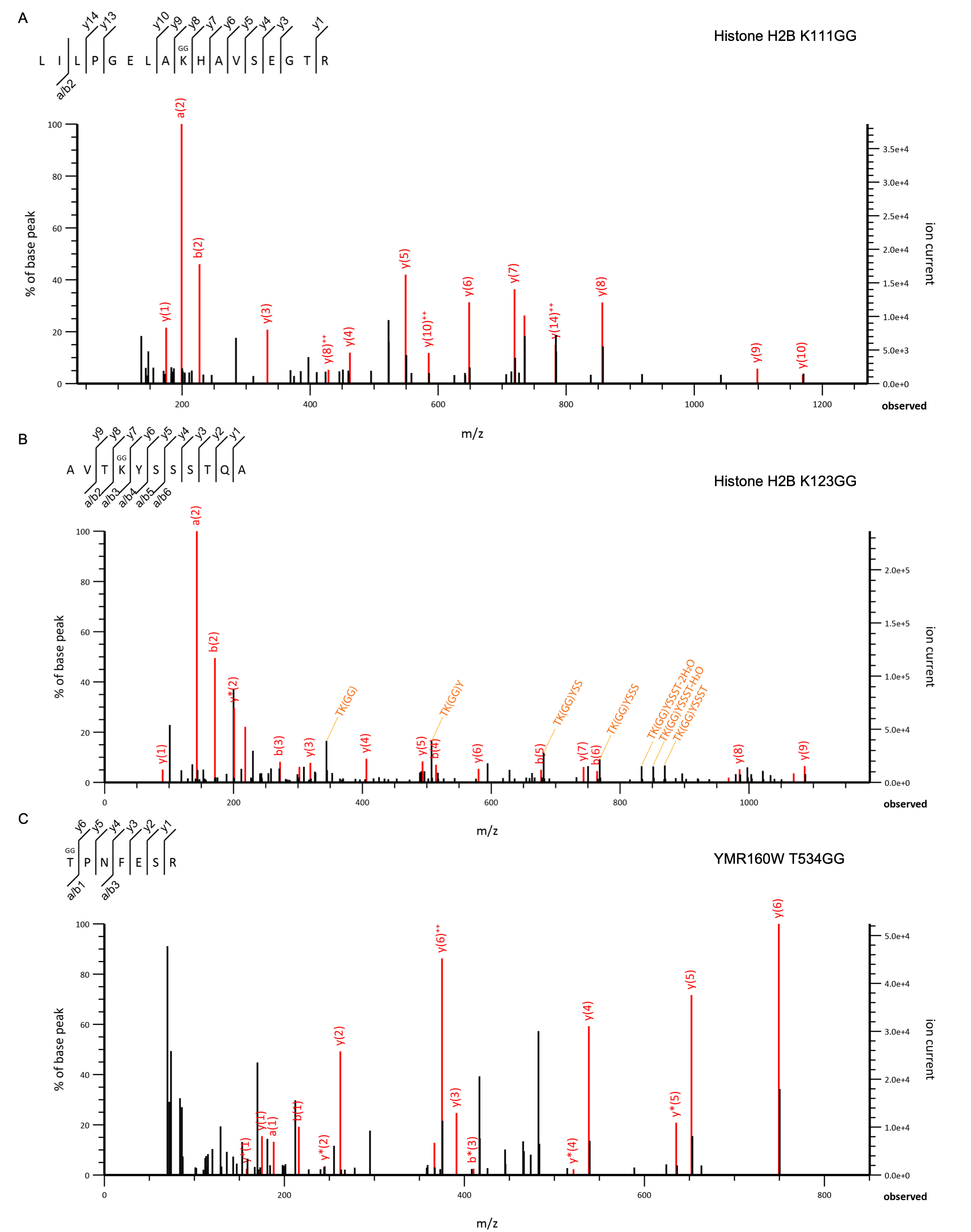

Supplement: S6 Fig — (A) Representative MS/MS spectrum of the Htb2 K111GG peptide. (B) Representative MS/MS spectrum of the Htb2 K123GG peptide. In addition to a/b/y ions, we identified multiple peaks from internal fragmentation and dehydration, potentially due to the serine/threonine-rich nature of the sequence. (C) Representative MS/MS spectrum of the YMR160W T534GG peptide. (TIF) [file pbio.3001501.s006.tif]
