# Supplementary material for: A versatile new tool derived from a bacterial deubiquitylase to detect and purify ubiquitylated substrates and their interacting proteins
Source: PLoS Biol. 2022 Jun 30;20(6):e3001501. doi: 10.1371/journal.pbio.3001501 (PMC9278747; doi:10.1371/journal.pbio.3001501)
Supplement: Attachment — Submitted filename: Zhang_etal_rebuttal.docx [file pbio.3001501.s010.docx]

We thank the reviews for their thoughtful and constructive reviews. Below we address all of the points they had raised.

Reviewer #1: This manuscript extends earlier work from the Hochstrasser lab by developing their previously described high-affinity ubiquitin (Ub) binding domain protein, OtUBD, into a convenient and effective reagent that can be used to capture (poly)Ub species from cell lysates or other complex mixtures. In particular, a covalently-linked resin-bound form OtUBD was shown to have several properties that make it very well-suited for applications in proteomics. Chief among these are the ability of OtUBD to capture monoUb protein conjugates as well as polyUb conjugates, and that either native or denaturing conditions can be used for binding and washing steps during affinity purifications. Although the use of OtUBD as an affinity reagent was perhaps obvious as a follow-up to the original Berk et al. 2020 paper, the methods and findings in this new paper by Zhang et al. are very useful; they are certain to be adopted widely by the very large number of researchers with interests
that involve Ub biochemistry.
Overall, this study was performed rigorously and the conclusions are supported by the experimental results. However, one exception (see item #1) and a few other issues are noted below.

1.     (p. 7 & Fig. 1D) The authors' concluded from the results in Fig. 1D that, relative to a single OtUBD, a 3xOtUBD tandem fusion protein binds Ub conjugates more efficiently. They speculate that avidity was likely responsible for the difference. However, that difference was seen only when the binding proteins were used at a density of 4.5 nmol/mL resin and disappeared when that was doubled (in fact, the MBP-OtUBD resin now looks like it bound more conjugates than the MBP-3xOtUBD form). A more likely explanation for the difference between the single and 3x OtUBD is that the latter resin had 3-fold more binding domain — i.e., the 4.5 nmol/mL resin with MBP-3xOtUBD had 13.5 nmol/mL OtUBD. The lanes that compared the 9 nmol/mL resins show that even doubling the amount of MBP-OtUBD dramatically increased conjugate binding. Avidity cannot be invoked to explain the greater binding of free monoUb, a monovalent ligand, by 3xOtUBD. Examination of the free Ub bands in the blot
suggests that the amount of Ub in the lysate exceeded the capacity of the OtUBD — but not 3xOtUBD — when used at 4.5 nmol/mL, and that doubling the OtUBD allowed almost all of the Ub to be captured.

We agree with the reviewer and have changed the corresponding text to reflect this. See line 146-148 in the revised manuscript.

2.    Pulldown of Ub species by OtUBD appears to capture polyUb conjugates with all types of Ub-Ub linkages, and quantification of the different linkages is in good agreement with an earlier report by Xu et al. 2009 [Note: Fig S4E should note the proper reference on the figure and in the legend]. This is important and useful information. However, the result could be interpreted by readers as suggesting that OtUBD will bind well to a Ub that has any one of its lysine(s) linked by another Ub. That was not shown; in fact, it is possible the some Ub-Ub linkages may interfere with OtUBD binding to a ubiquitinated (i.e., proximal) Ub in a polyUb chain. Nonetheless, because every polyUb chain would contain a distal Ub subject to tight binding by OtUBD, all Ub-Ub linkages would be recovered in the pulldown. It would be helpful if the authors would discuss this point in their paper and elaborate upon what's known and not known regarding linkage specificity of OtUBD.

We agree with the reviewer’s comments about the ambiguity regarding the basis of OtUBD’s apparently similar ability to bind Ub chains with different linkages. We added a brief discussion on this topic, including on the point about whether tight binding to the distal ubiquitin in a chain by OtUBD could allow similar binding to different chain types (Lines 409-418 in the revised manuscript).

3.    A very important consideration in the use of the OtUBD resin for pulldowns, and especially in the context of unbiased proteomics analyses, is that care must be taken to ensure that the OtUBD binder is used in excess over the total Ub. This point should be made in the paper.

Indeed, we did determine the amount of resin to use before our pulldowns used for proteomics analyses. We have added an additional sentence emphasizing this in our main text (Lines 337-338 in the revised manuscript).

4.    (Fig. 3C,F) In panels C and F, it would be helpful to identify the positions expected for free monoUb. It looks like free monoUb was captured efficiently using native conditions but not with denaturing conditions. Is that the case? If so, could that be because there is less functional OtUBD available in the denaturing condition? It would be informative to compare the two binding conditions with respect to the capacity of the OtUBD resin for monoUb.
We have changed Fig. 3C with a nicer looking blot as the image quality of free Ub in the original figure was not ideal. Overall, in our replicates of these experiments, the native conditions did not appear to capture more free Ub than the denaturing conditions, as shown in the updated figure. We have also adjusted Fig. 3F to show the free Ub without any cropping. We have found it technically difficult to always obtain high-quality bands for the free Ub in our Westerns, potentially due to loss of protein during membrane transfer due to its small size or variable refolding of Ub on the membrane. We find that the quality of the higher mass smear representing bulk ubiquitylated substrates is more reproducible than the free Ub bands in assessing binding to the various resins.

Some minor points:
5.    The structure of the 3xOtUBD (i.e., sequences of the linkers between the OtUBDs) should be included in the Methods or Supplementary Data.

We added the sequence in Supplementary Data 1.

6.    The Introduction includes too much review of the ubiquitin system.

We found it necessary to introduce the basic concepts of the ubiquitin system, especially to a general audience that may not have as much familiarity with this information, which is relevant later in the manuscript (e.g. DUBs; yeast with epitope-tagged ubiquitin; different ubiquitin enrichment methods; substrates of E3 ligases; different lysine linkages). We found it hard to trim down the Introduction too much but cut several extraneous phrases and sentences from the beginning section.


Reviewer #2: The manuscript by Zhang, et al., presents a method for purification of ubiquitylated proteins from total yeast and human cell extracts, suitable for combining with mass spectrometry approaches for identification of ubiquitylated proteins and their sites of modification.  This is a variation of methods that have been widely developed and employed over at least the last dozen years and is based on the use of isolated ubiquitin-binding domains (UBDs) from various naturally occuring proteins.  The innovation here is that the UBD used here (OtUBD) is from a bacterial effector protein with a particularly high affinity for ubiquitin (Kd of approximately 5 nM), as reported recently by the same group in Nature Communications (2020).  Some of the advantages claimed for this modified method are that 1) it is more efficient that other methods, 2) that it recognizes all seven ubiquitin linkages and 3) that it recognizes non-canonical (non-lysine-based) ubiquitin linkages.
All of these claims require further substantiation and the first two of these require quantitative analyses, which are currently lacking.  

The relevant statement on PLOS Biology standards for methods articles are that, "… the method presented may demonstrate substantial improvements to currently used methodologies. They would need to significantly outperform their predecessors by precision, resolution, speed, accessibility, and/or cost".  With this in mind, specific comments follow.

The claim that it the OtUBD matrix recognizes all types of ubiquitin chain linkages is based on identifying these in mass spec experiments using total yeast extracts.  This is a good start, but there should be some quantitative assessment of the relative affinity for different linkages types, for example using purified chains of somewhat uniform length.  These are of course readily available.  As a side note, an experiment using human cell extract did not identify one of the seven linkages, but then it is noted that the experiment was only done with a single replicate.  I am wondering why any mass spec experiment done with a single replicate is included in the manuscript, at all.

We have attempted quantitative comparison of affinities of OtUBD towards different linkage types. However, in initial ITC experiments that we performed with diubiquitin molecules with K48 and K63 linkages, we found that the affinity of OtUBD towards them was much higher than that towards free ubiquitin (in the sub-nanomolar range and reaching the theoretical detection limit of ITC), making it was hard to make accurate comparisons. Optimizing other methods to quantitatively comparing the affinity of OtUBD to ubiquitin polymers with different lysine linkages is costly, and the results in our opinion are not essential for the current work. Quantitative proteomics experiments, for example AQUA, will provide important information of OtUBD’s preferences towards endogenous ubiquitin substrates with different lysine linkages, but this type of experiment is beyond the scope of this study.

The proteomics analysis from our yeast studies provides strong evidence that OtUBD can enrich all seven lysine chain types. OtUBD could have a preference towards certain linkage types but because it is likely to bind to the unmodified distal ubiquitin with high affinity, when excess amount of resin is used, we would expect substrates with all lysine linkages to be enriched without strong biases. We have discussed this point in Line 409-418.

To address the Reviewer’s point about having had only one replicate with the human cell experiments, we have now added additional replicates to these proteomics analyses.

Related to the prior point, given that the entire premise of the method is that it is more efficient than other related methods (i.e., using different UBDs), some direct quantitative comparisons to a handful of the differing UBDs in use is in order.  While there are some experiments here that compare to the well-known TUBE matrix, even here it is entirely non-quantitative.

In response to this comment, we have now added the said comparisons with additional previously published UBD-based reagents (Dsk2 and the monomeric UBA of the TR-TUBE). This is presented in our revised Figure 4 and updated in the text (Line 235-319). In these new experiments, we have found that OtUBD outperforms these different UBD/TUBE-based reagents mainly in two aspects: 1) it can efficiently bind to mono-ubiquitylated substrates, and 2) it can be performed efficiently in denaturing conditions, which helps differentiate actual ubiquitylated substrates from ubiquitin-associating proteins.

To make quantitatively relevant comparisons between datasets, we made several additional efforts: 1) we made resins of different UBD/TUBE reagents using the same strategy as used for the OtUBD resin with equal molar amounts of bait proteins loaded on each resin; 2) we tested different amounts of resins (Fig. 4A,B); and 3) we used saturating amounts of resin for each of the comparisons we made in Fig. 4C-G. This has allowed us to better compare the different affinity reagents. For the comparisons shown, the relatively strong binding of OtUBD compared to the other affinity reagents to monoubiquitylated and oligoubiquitylated proteins is apparent. We hope these new data make clear the advantages OtUBD has for ubiquitin-conjugate enrichment over existing methods.

The claim that the method can detect non-canonical ubiquitin conjugates (e.g., ser/thr/cys linkages is not sufficiently discussed or tested.  At the very least this needs to be presented more completely and carefully.

Every type of ubiquitin conjugate contains a mono-ubiquitin or distal ubiquitin in a chain that is expected to bind to OtUBD with high affinity (discussed in Lines 416-418). This predicts that OtUBD can detect noncanonical ubiquitin conjugates. When we included noncanonical substrate protein linkages in our proteomics search algorithm, we found a handful of assigned identifications of noncanonical ubiquitylation. We agree with the Reviewer that these assignments need to be viewed with caution. We have manually examined a few of the spectra representing a noncanonical ubiquitylation assignments and reported one spectrum that we are confident to represent a threonine side chain ubiquitylation (Fig. S6C). We have worded our conclusion more carefully to indicate the high likelihood (but not certainty) that OtUBD can bind noncanonical ubiquitin modifications of substrates and added a note making clear the need to further validate any such hits (Line 466-470).

In summary, the potential advance here is technical and not conceptual.  As such, it is important to include additional quantitative assessments to substantiate the central claims.  


Reviewer #3: Zhang et al. "A versatile new ubiquitin detection and purification tool derived from a bacterial deubiquitylase"

In this paper, Zhang and colleagues characterize the functionality of OtUBD, an isolated ubiquitin binding domain from a bacterial deubiquitylating enzyme, as a ubiquitin enrichment reagent. This work extends the findings of a manuscript published last year by the same group which included extensive biochemical analyses of OtUBD, and a crystal structure of OtUBD bound to ubiquitin. Despite extensive successful efforts by the field to characterize the ubiquitylated proteome, our ability to reliably detect specific ubiquitylated proteins remains limited. In large part, this inability is due to the imperfect reagents (di-gly antibodies, TUBEs, epitope-tagged ubiquitin) currently available to enrich the ubiquitylated proteome from lysates. Here the authors demonstrate the ability of recombinant OtUBD to isolate ubiquitylated species from yeast and human cell lysates. They show that OtUBD protects ubiquitylated proteins from deubiquitylation after cell lysis. Additionally, the authors use OtuBD to purify ubiquitylated species under both native and denaturing conditions. Finally, they use OtUBD to monitor ubiquitylation on specific proteins, as well as globally survey the ubiquitylated proteome via mass spectrometry.

Zhang et al. nicely demonstrate the utility of OtUBD in ubiquitin enrichment. Specifically, the ability of OtUBD to bind monoubiquitylated substrates represents a significant advantage over the currently used TUBE-based reagents, which, as the authors note, do not have a high affinity for monoubiquitin. Thus, I think with some improvements, this study can have broad interest in the field who may want to try out an OtuBD-based enrichment strategy to study the ubiquitylation of, or by, a protein of interest.

Major comments:
1.    The authors do a fairly rigorous characterization of the OtUBD. However, they mainly compare its efficacy to a tandem ubiquitin binding entity (commercially available which I think is in an inappropriate comparison. The authors themselves make a tandem reagent with the OtUBD and find it less effective than the single domain reagent. Thus, the better and more appropriate comparison is to a reagent with a single UBA (the yeast DSK2-UBA is a commonly used ubiquitin affinity reagent, but there is a large list of potential single domain ubiquitin affinity regents that can be used (UIM, UBD, UBA, etc). Even if these are not commercially available, the authors certainly have the ability to produce one of these regents in a similar manner as the OtUBD. This is important for two reasons. First, the authors state the higher affinity of OtUBD as one of the characteristics that likely make it a better ubiquitin capture reagent. As such, comparison to the yeast DSK2-UBA (or other
single domain UBA affinity reagent) may reveal the OtUBD to bind much more ubiquitinated proteins and be a better reagent (compared to the current comparison with the TUBE). Second the authors make the specific point (in figure 1 and figure 4) that the OtUBD is better at binding (via protection in figure 1) mono-Ub proteins than the TUBE. This is expected given the previous studies showing that a single ubiquitin binding domain captures more mono-ubiquitinated proteins than tandem domains. Here the comparison to a single UBA (like the DSK2-UBA) is a more appropriate comparison. I recommend repeating some of these studies using this more appropriate comparison. Similarly, in figure 4B, the authors compare OtUBD to TUBE enrichments under native conditions but don't have a similar comparison under denaturing conditions where they point out a specific enrichment for a putative mono-Ub modified Rpt5. Having a TUBE-based and a single-UBA-based enrichment for comparison would be
useful to evaluate these results.

We thank the Reviewer for raising these valuable points. We originally focused on comparing OtUBD with a TUBE because TUBEs have been shown to be much more efficient in enriching bulk ubiquitylated species than monomeric UBAs (for example, in Hjerpe 2009). We would also like to clarify that we did not find the TUBE version of OtUBD less efficient than a single OtUBD (see updated Figure 1D and our response to Point 2 below). We decided it was unnecessary to use OtUBD in the tandem format and chose not to further develop the 3xOtUBD due to foreseeable pitfalls such as potential protein precipitation and difficulty in eluting ubiquitylated proteins from the resin (as we have seen with the TR-TUBE).

We see the Reviewer’s point about the importance of comparing OtUBD to other monomeric UBD-based reagents. To address this, we made a Dsk2 resin as well as a TR-UBA (the monomeric UBA of the TR-TUBE we previously used) resin using the same strategy we used for making the OtUBD and TR-TUBE resins. We then performed a series of pulldown comparisons with these different resins. (See the updated Figure 4 and Lines 235-319 for these new results.) (We did purify both full length Dsk2 and Dsk2-UBA for comparison and found that the Dsk2-UBA resin we made did not perform as well as the full-length Dsk2 potentially due to insufficient linker length. We decided to stick with the full length Dsk2 for our comparisons since full length Dsk2 was also used in other studies (e.g., Anindya et al, 2007).)

2.    In figure 1D, at the lower concentration of bait protein, the 3xUBD enriches ubiquitylated proteins more efficiently than the single UBD. However, the reverse is true with the higher concentration of bait protein. Why is this the case?

After repeating the experiments and looking at blots from different replicates, it became clear that this was most likely due to uneven loading/transfer in the said lanes. At lower concentration, UBD performed less well than 3xUBD, and at higher concentration, UBD and 3xUBD displayed similar efficiency. This is consistent across multiple replicates. We have switched the representative Western blot to avoid confusion.

3.    Because this is a methods development study, comparison to the current standards is important. I appreciate the proteomics characterization of proteins enriched using OtUBD as an affinity handle. The authors compare the results they obtained from yeast lysates to three other studies using di-Gly-based proteomics methods. It would also be useful to compare the results to other proteomics experiments using TUBE-based or other ubiquitin-binding domain affinity methods as that is the most similar to the OtUBD method investigated here. Similarly, when the authors performed a similar proteomic study using mammalian cell lysates, the authors compare their results to those obtained from a previous study using FK2-antibody-based enrichments from a study published in 2005. It would be useful to have some uniformity to the comparisons that are being done. I suggest the authors compare their mammalian proteomics results with di-GLY-based studies (similar to what they did with the yeast proteomics results, and there have been many such mammalian di-GLY proteomics studies in the last 10 years). Again, also comparing these results with recent mammalian-based TUBE or ubiquitin binding proteomics results, such as those reported for TR-TUBE itself in Yoshida et al 2015, or Lear et al. J Biol Chem (2020) would be the best comparison to the OtUBD results reported here. Making relevant comparison to more recent studies will be essential in evaluating any methodological gains from using the OtUBD-based enrichment strategy reported in this study.

One point that we realized through browsing the literature for different Ub methods is that the total number of ubiquitylated proteins detected reflected to a great extend the depth of peptide reads from the proteomics method rather than just the efficiency of the Ub enrichment method. Often, fractionation of the digested peptides with an orthogonal method prior to LC-MS/MS greatly increased the total identification especially in the case of human ubiquitylome, where the ubiquitylome is more complex than that of yeast. For that reason, we were cautious about comparing our results with data from the literature. The original comparison we did was to perform OtUBD pulldown and FK2 antibody enrichment side by side. The reason for choosing FK2 antibody was because it has been shown to be compatible with the urea denaturing pulldown conditions in Matsumoto 2005. We have added the suggested comparison to other proteomics results reported in literature and added a brief discussion (Lines 386-407 also Lines 528-547) to help in interpreting the comparisons.

4.    While OtUBD binds the high molecular weight ubiquitin "smears", it shows a clear preference for binding free ubiquitin in lysates. This is seen, for example, in the UBD FT lanes in Fig 2C, where free ubiquitin is depleted more completely than the high molecular weight species. If the unbound fractions were subsequently incubated with additional OtUBD, could a more complete enrichment be achieved? If not, it will be important to understand the nature of the ubiquitylated proteins that are not well captured by OtUBD.

We usually observe a light smear of ubiquitylated proteins in the flow through fractions. In most cases, the smear is very faint (see Fig. 2B, 3G, S3A, S4A, B). While this can vary from experiment to experiment, potentially due to imperfect measurement of total protein concentration, pipetting differences across experiments, etc., in the case of the pulldowns performed with His-Ub, the unbound smear appears to be slightly more prominent (we estimate that in the example shown in Fig. 3C, the flowthrough fractions contain between 8-20% of total ubiquitylated proteins present in the input). In a replicate of the His-Ub pulldown we performed recently, we ran the flowthrough over fresh OtUBD resin and saw a slight but not complete reduction in the overall intensity of the smear (see blot below, FT vs. FT2). The apparent inefficiency of the second capture may be due to the very low concentration of ubiquitylated-substrates present in the flowthrough or indicate that certain ubiquitylated substrates bind to the resins less readily due to their physical or biochemical properties, for example, ubiquitin modification or binding to other factors in the extract. Understanding the nature of these “leftover” ubiquitylated proteins can be interesting but it is technically very challenging. Overall, we have seen that OtUBD resin can very efficiently bind to ubiquitylated substrates across a wide molecular weight range.

5.    In supplemental figure 4E, the authors report total TIC as a box-and-whisker plot in which the relevant controls are not interpretable. It would be more useful to just show the overlaid total TIC chromatograph from each of the runs. As it is currently reported, I am not sure what the box or whiskers represent.

We switched Figure S4E (now S3E) to the suggested TIC chromatograph.

6.    In the supplemental table reporting the proteomic results, in table 2 reporting peptides with di-GLY modifications, there are some examples where the modification is listed on the terminal amino acid (A6ZYV5 for example with the ILENDLk peptide). This suggests that the data has been inappropriately processed as trypsin does not cut at ub-modified lysine residues. I suggest the authors re-process their data to not allow C-term di-GLY modifications as these are almost certainly false-positive identifications.

The Reviewer raises a good point. However, it was technically challenging for us as the Mascot software we use to search for di-Gly modified peptides lacks the option of excluding these C-terminal di-Gly assignments. We have therefore manually removed these assignments from our di-Gly peptide table and also added a note in our proteomics data pointing out the limitation of the search algorithm and emphasized the importance of manually validating hits of interest.

Minor comments:
1.    In line 285, the figure reference should be for 3A, not 4A.
2.    In line 330, the figure reference should be for S4G, not D.
3.    In line 337, the figure reference should be for S4H, not E.

Thank you! We have changed these typos.

4.    In line 339, there is no reference given for the HeLa proteomics data.

This is because the HeLa proteomics data from FK2 immunoprecipitation was obtained in our study (and we have now added two more replicates). (See our answer to main point 3.)

Reviewer #4: SUMMARY
In this manuscript, Zhang et al. sought to establish a novel tool to detect and purify ubiquitin by using a high-affinity ubiquitin-binding domain derived from a bacterial deubiquitylase from Orientia tsutsugamushi. For this purpose, the authors characterized the ubiquitylome and ubiquitin-associated proteome of yeast and compared ubiquitylated substrates from yeast and human samples to assess the efficacy of existing methods. In an initial screen of ubiquitylation protection and pulldown experiments, the authors show that OtUBD is capable of protecting ubiquitylated proteins from DUBs and of enriching these targets. The authors were able to generate a covalently-linked OtUBD resin for ubiquitylated protein purification and by introducing the V203D mutation in OtUBD, they further determined its binding affinity and specificity. Using the OtUBD pulldown in denaturing versus native conditions revealed the enrichment of proteins that are covalently bound to ubiquitin. Moreover,
the authors could demonstrate consistent results with OtUBD for human samples. In further pulldown experiments under distinct conditions and by using yeast strains which lack the ubiquitin E3 ligase Bre1 or the DUB Ubp8, the authors found that OtUBD resin can facilitate the detection of both, monoubiquitylated and polyubiquitylated proteins. The authors could verify this result in human cells. After establishing the method, the authors used OtUBD pulldown proteomics on native and denatured lysates from yeast and human cells. Interestingly, the authors found much more enriched ubiquitylated proteins in comparison to results obtained from immunoprecipitation using FK2. Lastly, the authors showed that OtUBD and label-free quantification facilitates the identification of potential E3 substrates which was challening in the past due to the instability and low abundance of ubiquitylated proteins and the transient nature of E3-substrate interaction. Moreover, this approach could
have great potential for the identification of substrates of other ubiquitin-related enzymes. Overall, the study of Zhang and colleagues clearly expands the methodological toolbox for exploring and analysing monoubiquitylated and polyubiquitylated substrates. There are only a few minor issues that the authors should consider.

1) Is there any explanation why there is more binding of ubiquitylated substrates with a higher bait protein concentration in the MBP-UBD than in the MBP-3xUBD as it is the opposite for a lower concentration?

After repeating the experiments and looking at blots from different replicates, it was clear that this is most likely due to uneven loading/transfer in the said lanes in the blot shown. At lower concentration, UBD performed less well as 3xUBD, likely due to saturation of UBD binding capacity. At higher concentration, both UBD and 3xUBD displayed similar efficiency. This is consistent across multiple replicates. We have switched the representative Western blot to avoid confusion.

2) Is there any deeper meaning of the striking band in the flow through of the native sample in the anti-ubiquitin blot of fractions from Co2+ IMAC?

The Reviewer raised an interesting point. We looked at all of our replicates for this experiment, the band did show up in all of them, though sometimes not as strikingly. We are not sure what the identity of the band is. It could be a species that cross reacts with the ubiquitin antibody. We have made a note in the figure legend on this.

3) The authors should perform at least three biological replicates for their analysis. Especially for their work in HeLa cells. Currently, they seemed to have done only one experiment in this setting.

We thank the Reviewer for pointing that out. We have added two biological replicates for the HeLa experiments.

4) Please make sure that the labeling in the figures is clear and consistent (e.g. under regard of upper- and lower case).

We have made the suggested changes.

5) Please make sure that every IB panel carries a complete molecular weight marker labeling.

Thank you. We have made the changes.
