# Supplementary material for: A versatile new tool derived from a bacterial deubiquitylase to detect and purify ubiquitylated substrates and their interacting proteins
Source: PLoS Biol. 2022 Jun 30;20(6):e3001501. doi: 10.1371/journal.pbio.3001501 (PMC9278747; doi:10.1371/journal.pbio.3001501)
Supplement: Attachment — Submitted filename: Answers_to_reviewer3_comments_3.docx [file pbio.3001501.s011.docx]

Reviewer #3: The revised manuscript has addressed the majority of our concerns. We would request the author clear up a few outstanding issues prior to publication (we do not need to see another revision).

1. We appreciate adding the direct comparison of OtUBD to the full-length DSK2 (it was a little odd that the authors used full length over the isolated UBA and claimed the full-length worked better given the full DSK2 protein will have a Ub fold at the N-term which can interact in trans with the UBA domain likely impairing ub binding in the lysate).  In new figure 4, under native conditions, OtUBD and Dsk2 seem to isolate different ubiquitylated Rpb1 species (the smear vs the triplet bands in Figure 4F). It is beyond the scope of this study to identify the various species, but we would request that the authors acknowledge and comment on this difference in the main text.

We have added in our main text a brief mention of the potential difference in isolated ubiquitylated Rpb1 species as the reviewer requested. However, we think the difference in the appearance of the bands (smear vs. distinct bands) could simply be a result of different protein amounts that were pulled down. It would be hard to tell if OtUBD and Dsk2 isolate distinct ubiquitylated Rpb1 species without further analysis, which as noted by the reviewer is beyond the scope of this study. We did notice that while Dsk2 and OtUBD performed similarly in purifying bulk higher molecular mass Ub species in general (as shown in Fig. 4A), it appeared to be much less efficient here in enriching ubiquitylated Rpb1 species. The identity of ubiquitylated Rpb1 species may play a role here.

2. It would be useful to see a silver stain or coomassie stain from the comparative pull down experiments to show that equal amounts of the OtUBD, DSK2, TR-UBA, TR-TUBE are present in the pulldowns. This will help validate the usefulness of the cross comparison (what if there is 4x less DSK2 on resin).
The resins have the bait proteins covalently attached, so there were no free bait proteins present in the pulldowns. We have a Coomassie-stained protein gel (see below) showing that when making the Dsk2, TR-UBA and TR-TUBE resins, similar amounts of proteins were added to the SulfoLink resins and the proteins were efficiently coupled to the resins (disappearance of band in the flowthrough from SulfoLink resin). The same analysis for OtUBD (see below) was not in the same gel (because that resin was made at an earlier time), but the TR-TUBE (same protein prep) was used in both earlier and later batches. The amount of each protein added to make the resins were reported in the Methods section. While there is likely some variability in the measurement of protein concentrations, it is very unlikely that there would be 4x less Dsk2 on the resin.

3. Last, we have some concerns about how the mass spec data, particularly how the ub-modified peptides were analyzed. The authors say they manually removed all identifications with C-term GG modifications. While this is useful in that those are certainly incorrect identifications, this will also alter the false discovery rate for the modified peptides.

The presence of those false positives in dataset indicate that many other false positives are present in the dataset that cannot be simply identified and removed as they did for the c-term modified peptides. In fact, it is entirely unclear how the modified peptides were identified and processed as the methods simply state that "GG modified peptides were further analyzed using
Scaffold PTM 3.3 software". The authors also suggest that Mascot is incapable of filtered search outputs with C-term modifications. There are at least two publicly available MS processing suites (Maxquant being one of them) that are capable of processing these modified peptides correctly.

It very unclear how the authors controlled the false-discovery rate for K-modified, or even more importantly, T or S di-gly modified peptides. When calculating the false-discovery rate, the number of incorrect matches (usually reversed peptides) that also have the K-GG or S-GG need to be considered separately from the unmodified peptides. How many reverse matches have S-GG or T-GG? How does that compare to the forward matches reported in their tables?

My guess is that the calculated FDR, when considering modified peptides separately, will be MUCH higher than is reported here. This is very important when reporting these peptides because if their identification is equally likely to be a false positive, there is no value in reporting those identifications (in fact that are as likely to be wrong as they are right). Mascot and certainly Maxquant will be able to control FDR for modified peptides only.

The reviewer raises valuable points here. We are aware of the challenges regarding the identification and validation of GG-modified peptides and acknowledge there are indeed limitations in our reports of GG-modified peptides. Redoing all searches for GG-modified peptides using a another algorithm such as Maxquant, which allows for the exclusion of GG-modification on a peptide C-terminus, may improve the overall confidence of the GG-peptides identification, but the extra time and costs made it beyond the scope of this methodology paper, which focuses on the new enrichment method for ubiquitylated substrates. We validated the enrichment methods by various approaches, including both biochemical and proteomics analyses. GG-modified peptides only constitute a very small portion of all identified peptides in our proteomics experiments, and we reported the GG-modified peptides identified by the search algorithm only as a reference for readers who may be interested in looking into some of the potential ubiquitylation substrates/sites further. We have emphasized in the main text and in S2 Data (previously named Supplementary Data2) that these identifications require further validation. To help clear up any confusion, we have made this emphasis clearer and added details to the proteomics methodology we used in our revised manuscript, both in the main text and in the supplementary data.

While we were unable to exclude peptide C-terminal K-GG assignments with the Mascot search algorithm and instead manually removed these entries, we noted that the vast majority of such mis-assignments happen when there are multiple K residues close to each other within the same peptide ,and the algorithm could not definitively assign the location of the GG site within the peptide based on the MS/MS fragmentation data. (A very common case are peptides ending with KK, KxK or KxxK.) In these cases, the C-terminal GG assignment was listed as one of the possible GG-site assignments on the same peptide. A C-terminal GG assignment without an alternative GG-site assignment within the same peptide is rarer. Overall, the false positives arising from C-terminal K-GG accounted for 6.2% to 7.5% of all GG-assignments in our experiments. We did observe a handful of C-terminal K-GG assignments that map to the end of a protein sequence; these were not manually removed since they are plausible.

The false discovery rates in all of our MS/MS identifications were controlled by standard target-DECOY analysis. After searching for protein/peptide identifications with Mascot, the results were loaded into Scaffold 5 software where a filter was applied to each dataset with an overall protein probability threshold of 99% with a minimum of 2 peptides and a peptide probability threshold of 95%. Any data entry not satisfying these criteria were not included. The filtering results in an overall protein FDR in the range of 0.1 – 0.7% for our three experiments and an overall spectra FDR in the range of 0.01 – 0.05%. For GG modified peptides specifically, the FDR is generally higher than the overall peptide/spectra FDR, which is as expected, and ranges from 0.08 – 4.51%. We have now added the FDR information for each individual experiment in the revised S2 Data (Supplementary Data 2) for readers’ reference. While Scaffold did not allow us to compare the FDR for K-GG versus non-canonical GG identification, further analysis of the Macot searches did confirm, as the reviewer suggested, that the FDR on the non-canonical sites is about 3 times of that for lysine modified sites, which put them in the estimated range of up to 15%. We have added this caveat to the notes in our supplementary document S2 Data.

We initially loaded the data filtered by the above-mentioned protein and peptide threshold settings in Scaffold into the Scaffold PTM 3.3 software to utilize their built-in site localization algorithm to help us differentiate between multiple potential GG modification sites located in the same identified peptides, which appeared in a subset of the GG-modified peptides we obtained after the filtering step in Scaffold. The software re-analyzes MS/MS spectra of modified peptides and calculates an Ascore value to predict site localization probabilities. We noted that this algorithm, which was originally developed for protein phosphorylation site analysis, may not be best suited for our experiments. To avoid the confusion this extra step of analysis may have caused, we omitted it in our revised S2 Data (Supplementary data 2) and instead directly reporting GG-modified peptide identified by Mascot that have been filtered by Scaffold.

While some of the non-canonical GG assignments involve peptides with both an internal K residue and an S/T/C residue, a number of the S/T/C-GG peptides identified by Mascot contain no K residues. These data, of course, did not indicate definitive S/T/C-GG identification but rather suggest of the possibility of detecting non-canonical GG-modification with our enrichment method. We have manually inspected a number of individual spectra identified to be S/T/C-GG modified and of these spectra, we were confident that one of them (reported in Fig. S6C) likely represents a real T-GG modification. Most other spectra did not provide the necessary fragments to confidently assign the modification site. As mentioned before, we have clarified our emphasis that all reported GG-peptides should be further validated in our revised manuscript to avoid any confusion.

In summary, we thank the reviewer for raising these critical points and have added more details in our supplementary data as well as method sections to clarify the methods we used.
